# Action of Extracellular Proteases of *Aspergillus flavus* and *Aspergillus ochraceus* Micromycetes on Plasma Hemostasis Proteins

**DOI:** 10.3390/life11080782

**Published:** 2021-08-02

**Authors:** Alexander A. Osmolovskiy, Laura Schmidt, Anastasia V. Orekhova, Sergey K. Komarevtsev, Valeriana G. Kreyer, Sergey V. Shabunin, Nikolay S. Egorov

**Affiliations:** 1 Biological Faculty, M.V. Lomonosov Moscow State University, 199234 Moscow, Russia; anastasia.orekhova@uniroma1.it (A.V.O.); vkreyer@yandex.ru (V.G.K.); 2 All-Russian Scientific Research Veterinary Institute of Pathology, Pharmacology and Therapy, 394087 Voronezh, Russia; skomarevtsev@yandex.ru (S.K.K.); svshabunin@rambler.ru (S.V.S.); 3 Institut für Agrar- und Ernährungswissenschaften, Martin-Luther-University Halle-Wittenberg, 06108 Halle, Germany; laura-schmidt.96@gmx.de; 4 Department of Public Health and Infectious Disease, “La Sapienza” University of Rome, 00185 Rome, Italy; 5 International Biotechnological Center, M.V. Lomonosov Moscow State University, 199234 Moscow, Russia; nsegorov21@mail.ru

**Keywords:** *Aspergillus*, extracellular proteases, fibrinolysis, thrombolysis, plasma coagulation

## Abstract

In this study, we investigated the properties of proteolytic enzymes of two species of *Aspergillus*, *Aspergillus flavus* 1 (with a high degree of pathogenicity) and *Aspergillus ochraceus* L-1 (a conditional pathogen), and their effects on various components of the hemostasis system (in vitro) in the case of their penetration into the bloodstream. We showed that micromycete proteases were highly active in cleaving both globular (albuminolysis) and fibrillar (fibrin) proteins, and, to varying degrees, they could coagulate the plasma of humans and animals (due to proteolysis of factors of the blood coagulation cascade) but were not able to coagulate fibrinogen. The proteases of both *Aspergillus* fully hydrolyzed thrombi in 120–180 min. Micromycetes did not show hemolytic activity but were able to break down hemoglobin.

## 1. Introduction

The genus *Aspergillus* is among the most abundant and widely distributed organism on earth, and at the moment, comprises 340 officially recognized species [1]. 

The incidence of *Aspergillus* infection has increased over the last few years due to the use of immunosuppressive and immunomodulatory drugs. It continues to cause significant morbidity and mortality worldwide, especially in immunosuppressed patients [2].

Micromycetes of the genus *Aspergillus* can secrete, into the environment, a large number of various proteins that make up their exoproteomes. These proteins alter the processes of signal transmission in the body, act as virulence factors in pathogenic processes, and affect the regulation of growth in a new environment [3]. The adaptation of *Aspergillus* to changing environmental conditions manifests itself by switching at the morphogenetic and physiological levels. This leads to the formation of a particular set of protein characteristics for a momentary environment. The composition of the *Aspergillus* exoproteome depends on many factors, including the growing conditions, the variety of nutrients present in the medium, and the composition of the medium [4,5]. Extracellular proteins are the main products of fungal metabolism involved in interactions between fungi and the host organism in pathogenic processes [6]. Recently, more and more information has supported that the exoproteome of *Aspergillus* contains proteins that are important factors of virulence, for example, gliotoxin and various hydrolases (especially proteases) [7]. Secretory proteins are secreted during conidia germination and hyphal growth, which help the growing filamentous fungus to overcome the host organism’s innate immunity and to create an unfavorable environment for its survival in aspergillosis [8]. Proteins of extracellular virulence of fungi promote the penetration of hyphae into the tissues of the host organism and, in combination with an induced inflammatory response, the fungus leads to tissue destruction [9].

*Aspergillus* exoproteome proteins circulating in the human body can act as biomarkers with an important potential role in the rapid diagnosis of aspergillosis and targeted therapeutic development. To date, the mechanisms of the pathogenic action of *Aspergillus* proteases on humans have been poorly understood [10]. However, the therapeutic potential of aspergillosis is limited, because fungal diseases are difficult to treat, since both the pathogen and the host organism are eukaryotes. This imposes restrictions on the use of antibiotics due to their low efficiency and increased toxicity to the host [11]. Azole drugs are currently the basis of anti-aspergillus therapy [12]. Overcoming this colossal challenge requires a systemic understanding of it, new therapeutic strategies, and a new diagnostic technology that can quickly identify *Aspergillus* while assessing drug resistance [13]. Studies on secretory proteins of *Aspergillus* are beneficial for creating these types of diagnostic or therapeutic developments [14].

Proteolytic enzymes as exoproteins are one of the proven factors of virulence of *Aspergillus* [15]. Currently, the results of a few in vitro studies of *Aspergillus* proteases, both pathogenic and non-pathogenic species, are known, which show that these enzymes can have different effects on the proteins of human tissues, depending on their substrate specificity. Thus, proteases of some *Aspergillus* can exhibit fibrinolytic, plasma-coagulant, and anticoagulant effects [16,17], and can initiate allergy and inflammation [18]. Due to their properties, these diagnostic proteases can be used for medical and veterinary purposes. Therefore, further study of the proteolytic enzymes of *Aspergillus* as extracellular virulence factors is required.

Here, we aimed to study the effect of micromycetes *A. flavus* 1 (with a high degree of pathogenicity) and *A. ochraceus* L-1 (a conditional pathogen) proteases on protein components of the hemostatic system.

## 2. Materials and Methods

### 2.1. Fungal Strains

Two strains of micromycetes, *Aspergillus flavus* 1 and *Aspergillus ochraceus* L-1, from the collection of filamentous fungi, producers of protease protein effectors of the hemostasis system (Microbiology Department, M.V. Lomonosov Moscow State University, Russia) were used.

### 2.2. Proteolytic Potential Determination

The strains of both micromycetes were grown on skim milk agar (SMA), plasma agar (PA), and blood agar (BA). PA and BA as basal components of media contained (in %) tryptone 0.7, peptone 1.0, yeast autolysate 0.5, NaCl 0.5, Na_2_CO_3_ 0.03, and agar 2.0, at pH 7.0–7.4, and additionally 8% sheep blood or 5% lyophilized sheep plasma (Sigma-Aldrich, Saint-Luis, MO, USA), respectively. The composition of SMA was (in %): skim milk powder (Sigma-Aldrich) 5.0, agar 3.0, at pH 6.0–6.5. Cultivation of micromycetes was carried out in Petri dishes, inoculation was performed by inoculating into the center of each medium in a Petri dish. After 5 days at 24 °C and 28 °C of inoculation, a hydrolysis of substrates around the colonies was visualized.

### 2.3. Submerged Cultivation and Proteases Preparations Obtaining

Cultures of *Aspergillus* were cultivated under submerged conditions in an orbital shaker ES-20/60 (Biosan, Riga, Latvia) for 5 days at 200 rpm, at 28 °C, in two consecutive stages, with growth on seeding (composition (in %): wort 6.7, glucose 1.0, and peptone 0.1, at pH 5.5–6.0) and fermentation media (composition (in %): glucose 3.5, fish flour hydrolysate 1.0, NaCl 0.2, starch 0.125, peptone 0.1, KH_2_PO_4_ 0.05, and MgSO_4_ 0.05, at pH 5.5–6.0). Then, the inoculum obtained via spore flushing with seeding medium was introduced into the seeding-culture medium and cultured for 2 days. Then, part of the biomass was transferred to the fermentation medium and cultured for another 2 days. The cultivation was carried out in 750 mL shake flasks containing 100 mL of culture medium [19]. The preparation of extracellular micromycete proteases were obtained by precipitation of proteins of the filtrate of the culture liquid with ammonium sulfate at the rate of 608 g (NH_4_)_2_SO_4_ per 1 L of sample. Salted out proteins were precipitated by centrifugation at 15,000× *g* for 20 min, at 4 °C. The resulting precipitate was dissolved in 0.01 M Tris-HCl buffer (pH 8.2) and dialyzed in dialysis tubes against the same buffer at 4 °C, for 18 h. The dialyzed preparation was frozen with liquid nitrogen and lyophilized (24 h). Fractionation of the obtained preparations (25 mg/mL) was carried out using preparative isoelectric focusing according to the Vesterberg method in a pH gradient of ampholines 2.5–10 and a sucrose density gradient of 0–40% in a 110 mL column (LKB, Bromma, Sweden) at a voltage of 800 V for 36 h [20]. The homogeneity of the isolated proteases was tested electrophoretically under native (nonreducing) conditions using the Davis method [21].

### 2.4. Fibrin Zymography

For fibrin zymography 0.12% (*w*/*v*) fibrinogen and 100 µL of thrombin (10 IU) were mixed in 12% polyacrilamide gel without SDS adding [22,23]. Electrophoresis was run in native (nonreducing) conditions at 12 mA and room temperature with directional ventilation. After stopping the electrophoresis, the gel was gently moved in 50 mM Tris-HCl, pH 8.0, containing 2.5% (*v*/*v*) Triton X-100 for 30 min at room temperature, and then washed for 30 min in distilled water and incubated for 18 h at 37 °C in zymogram reaction buffer consisting of 0.02% (*w*/*v*) NaN_3_ and 30 mM Tris-HCl, pH 8.0. Finally, gel was stained with Coomassie blue R-250 standard solution for 2 h and washed 3 times with 7% (*v*/*v*) acetic acid. Clear bands were detected as fibrin hydrolysis areas.

### 2.5. Clotting Assay

The ability to coagulate blood plasma of proteases was studied with 0.4% (*w*/*v*) solutions of human and bovine fibrinogen (Sigma-Aldrich, Saint-Luis, MO, USA) and human and rabbit plasma (Renam, Moscow, Russia), non-diluted and diluted 2 times. In both cases, 100 μL of the sample was added to 200 μL of substrate solution and incubated under static conditions 10 min at 37 °C, and 0.1% (*w*/*v*) thrombin (Sigma-Aldrich) was used as a positive control. After incubation, the clotting of substrates was visualized.

### 2.6. Determination of Proteolytic Activity

The proteolytic activity of fungal proteases was determined with 1% (*w*/*v*) suspensions of Hammerstein’s casein (Sigma-Aldrich), human serum albumin (Sigma-Aldrich), bovine serum albumin (Sigma-Aldrich), and horse hemoglobin (Reanal, Budapest, Hungary) by Anson–Hagihara’s modified method [24]. For the reaction, 200 μL of the sample and 400 μL of suspension of the corresponding protein substrate prepared in 0.1 M Tris-HCl buffer (pH 8.2) were incubated for 10 min at 37 °C, with permanent shaking (600 rpm). The reaction was stopped by 600 μL of 10% trichloroacetic acid. Then, A_275_ was measured in supernatant after sample centrifugation (12,400× *g*, for 10 min). The activity was expressed in micromoles of tyrosine formed in 1 min in 1 mL of culture liquid (U_Tyr_).

### 2.7. Study of Fungal Proteases Effect on Different Deficient Plasmas

Determination of the activator activity of the proteases was performed with chromogenic peptide substrate Z-D-Arg-Gly-Arg-pNA (S2765, Chromogenix, Milan, Italy) after preliminary incubation with normal donor plasma and plasmas lacking factor II, factor XI, and factor XII (Renam). For the reaction, 200 μL of a sample were mixed with 50 μL of corresponding plasma and incubated with shaking (600 rpm) for 5 min at 37 °C. After that, 100 μL of 0.05% solution of a chromogenic substrate in 0.05 M Tris-HCl buffer, pH 8.2, were added and incubated for 5 min under the same conditions. The reaction was stopped by the addition of 200 μL of 50% acetic acid [16]. Activity was expressed in units per mg of the protein (U/mg).

### 2.8. Thrombolysis Studies

Thrombolytic activity of proteases was measured gravimetrically as described by Kotb [25]. To prepare thrombi in suspended Eppendorfs, 200 μL of plasma and 20 μL of 0.1% (*w*/*v*) thrombin (Sigma-Aldrich) were mixed and stored for 30 min at 37 °C, after which they were reweighed with a stabilized thrombus. Then, 250 μL of the sample was added and incubated with stirring (250 rpm) at 37 °C for 30, 60, 90, 120, and 180 min. Partial liquefaction of the thrombus and its separation from the Eppendorf walls were observed. The lysate was carefully removed using filter paper and the dry residue was weighed together with the Eppendorf, after which the % of non-hydrolyzed thrombus was calculated.

### 2.9. Protein Determination

The protein content was determined by the Bradford protein assay [26]. For this purpose, 950 μL of the Coomassie Brilliant Blue G-250 reagent were added to 50 μL of the sample and A_595_ was recorded.

Reactions were carried out in a TS-100 thermoshaker (BioSan). The optical density of the solutions was measured on an Eppendorf kinetics spectrometer (Eppendorf, Hamburg, Germany).

Purified fungal proteases were used in the physiological range in all studies, for each reaction the enzymes concentration was selected in accordance with the data available in the literature [27].

Each experiment was carried out in three replicates, the error of the mean did not exceed 5–7%. All data were statistically processed using MS Excel 2019 and STATISTICA 7.0. Each experiment carried out in triplicate was subjected to analysis using the Mann–Whitney U test. Differences were considered to be statistically significant at *p* < 0.05.

## 3. Results and Discussion

In this study, an integrated approach was used to study the effect of proteases of microscopic fungi *A. flavus* 1 and *A. ochraceus* L-1 on the components of the hemostasis system. In the first stage, the possibility of the growth of both micromycetes on media containing protein substrates, i.e., casein, blood plasma, and blood itself, was studied at three temperatures (24, 28, and 37 °C). In the second stage, proteases were isolated from the culture liquid of *Aspergillus* and their ability to hydrolyze bloodstream proteins, i.e., hemoglobin, albumin, and fibrin (thrombi), was assessed.

**Figure 1:**
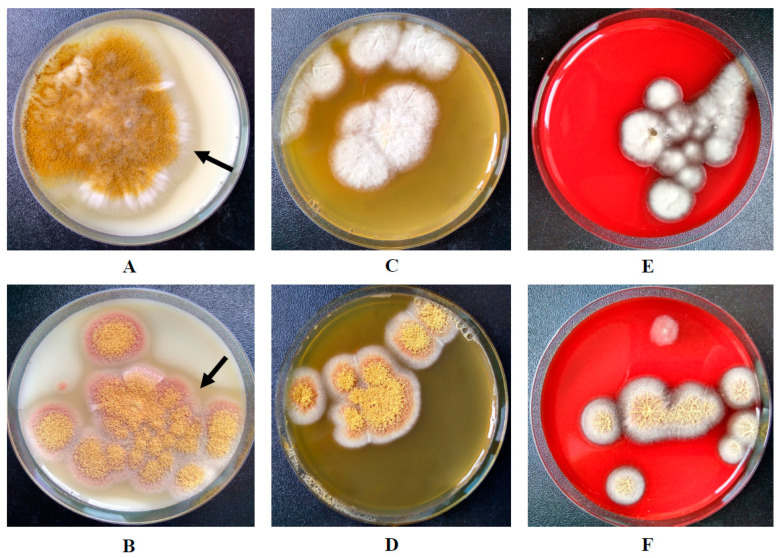
Growth of *A. flavus* 1 on: (**A**) SMA; (**C**) PA; (**E**) BA. Growth of *A. ochraceus* L-1 on: (**B**) SMA; (**D**) PA; (**F**) BA. The arrows show the zone of casein hydrolysis around the micromycete colonies.

The micromycetes, *A. flavus* 1 and *A. ochraceus* L-1, both showed optimal growth at a temperature of 28 °C. At 37 °C, growth was practically not recorded on any of the media used, which indicated the limited germination of the fungal strains used in the work at physiological temperature in the human body. At 24 °C, the growth was not great, the growth rates of *A. flavus* 1 were 4.3 ± 0.2 mm per day on SMA, 3.8 ± 0.4 mm per day on PA, and 4.0 ± 0.4 mm per day on BA. *A. ochraceus* L-1 demonstrated growth rates of 4.0 ± 0.3 mm per day on SMA, 3.9 ± 0.2 mm per day on PA, and 3.9 ± 0.2 mm per day on BA. At 28 °C, the growth rates were slightly more. For *A. flavus* 1, the growth rates were 5.4 ± 0.5 mm per day on SMA, 5.0 ± 0.3 mm per day on PA, and 5.1 ± 0.2 mm per day on BA. For *A. ochraceus* L-1 these growth rates were 5.1 ± 0.4 mm per day on SMA, 4.9 ± 0.5 mm per day on PA, and 5.0 ± 0.2 mm per day on BA. Micromycetes were found to be incapable of showing hemolytic activity when growing on BA (Figure 1).

Visual hydrolysis of PA was also not observed. The SMA, where the zone of substrate hydrolysis was clearly manifested, served as a marker of the ability to secrete proteases by both strains (Figure 1). With growth of *A. ochraceus* L-1 on media, it released a red-brown pigment.

**Figure 2:**
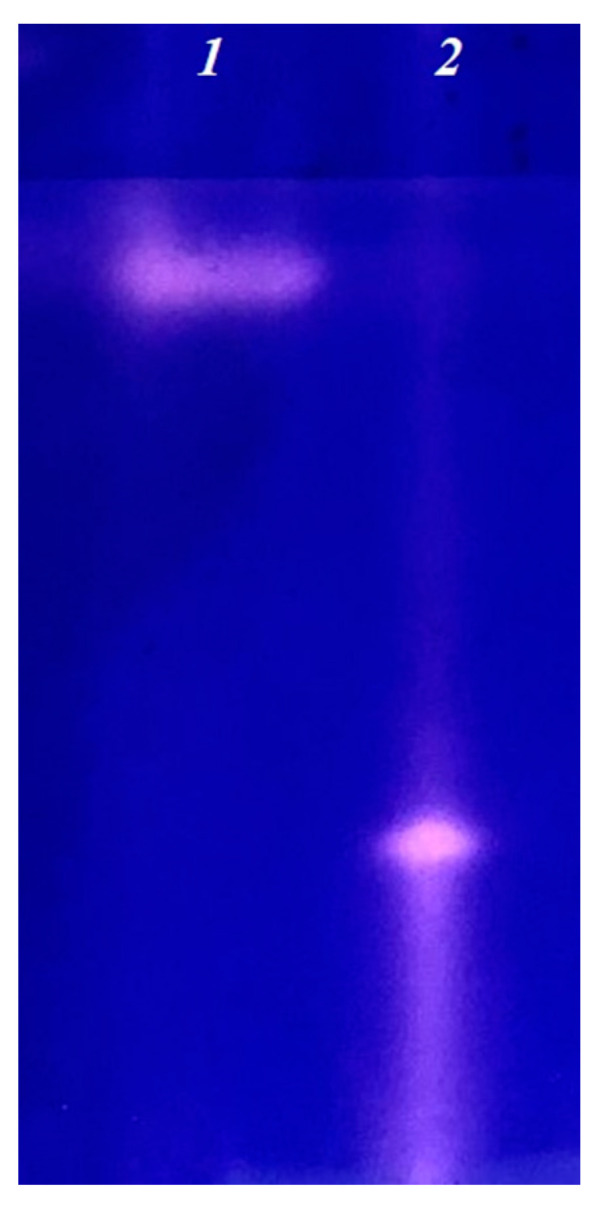
Fibrin zymogram of extracellular proteases of *A. flavus* 1 (**1**) and *A. ochraceus L-1* (**2**).

Despite the absence of significant results in the manifestation of pathogenicity of micromycete proteases during fungal growth on the differential media used, protease preparations were obtained using submerged cultivation. Their proteolytic activity was revealed using fibrin zymography. Figure 2 shows that the preparations of micromycetes, *A. flavus* 1 and *A. ochraceus* L-1, contain proteases that actively hydrolyze fibrin and differ in electrophoretic mobility. Fibrin zymography has shown that proteases of both strains have a direct effect on fibrin.

Proteases were isolated by preparative isoelectric focusing for further in vitro studies. The homogeneity of the obtained proteases was confirmed by electrophoresis (data not shown).

One of the activities of micromycete proteases in relation to the components of the hemostasis system is the clotting of blood plasma and fibrinogen. Proteases *A. flavus* 1 and *A. ochraceus* L-1 were tested with both animal and human samples. It was shown that, neither protease *A. flavus* 1 nor protease *A. ochraceus* L-1 had the ability to coagulate both human fibrinogen and bovine fibrinogen. Negative results were obtained when studying the coagulation of rabbit plasma by proteases of both strains of micromycetes. In the case of human plasma, protease *A. ochraceus* L-1, in contrast to protease *A. flavus* 1, coagulated human plasma both without dilution, and diluted in two times plasma. These results confirm our earlier data on the ability of protease *A. ochraceus* L-1 to activate factor X in human blood plasma [28].

The study of the hemoglobinolytic and albuminolytic activities of both proteases showed that they were capable of hydrolyzing both substrates to varying degrees. The results obtained are presented in Table 1. 

Thus, protease *A. flavus* 1 had greater hemoglobinolytic activity and albuminolytic activity with human albumin. Protease *A. ochraceus* L-1 had the highest albuminolytic activity with bovine albumin. Thus, both proteases, *A. flavus* 1 and *A. ochraceus* L-1, if they enter the bloodstream, can cause uncontrolled destruction of globular proteins, primarily albumin, as well as hemoglobin in the presence of destroyed erythrocytes.

The next stage of the study was to investigate the thrombolytic activity of the isolated proteases. For this, we assessed the lysis ability of newly formed thrombi.

**Figure 3:**
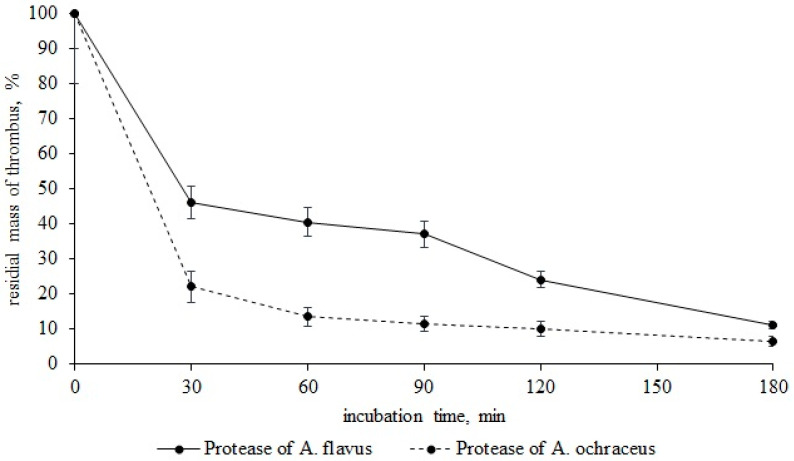
Thrombus lysis (residual mass, %) by proteases *A. flavus* 1 and *A. ochraceus* L-1.

As seen from Figure 3, proteases *A. flavus* 1 and *A. ochraceus* L-1 dissolve blood clots rather quickly. Therefore, after 30 min of incubation of a thrombus with protease of *A. flavus* 1, the residual mass of a thrombus was 46.2%. Incubation of a thrombus with incubation with protease of *A. ochraceus* L-1 was 22%. In both cases, the thrombus was hydrolyzed by 70% in 90 min. After 180 min of incubation, the residual masses of the thrombus with proteases *A. flavus* 1 and *A. ochraceus* L-1 was 11 and 6.4%, respectively. In view of the fact that the proteases of these micromycete strains previously showed fibrinolytic activity, it is obvious that thrombus fibrin is hydrolyzed directly under their action, and this is what leads to the dissolution of thrombi [29,30]. Thus, in addition to globular proteins, proteases of both micromycetes can also hydrolyze fibrillar proteins of the bloodstream, i.e., we can talk about their complex effect on the proteins of the hemostasis system. This means that when *Aspergillus* spores enter the bloodstream, they may secrete proteases for the use of human proteins present in the bloodstream as nutrient substrates [31,32].

**Figure 4:**
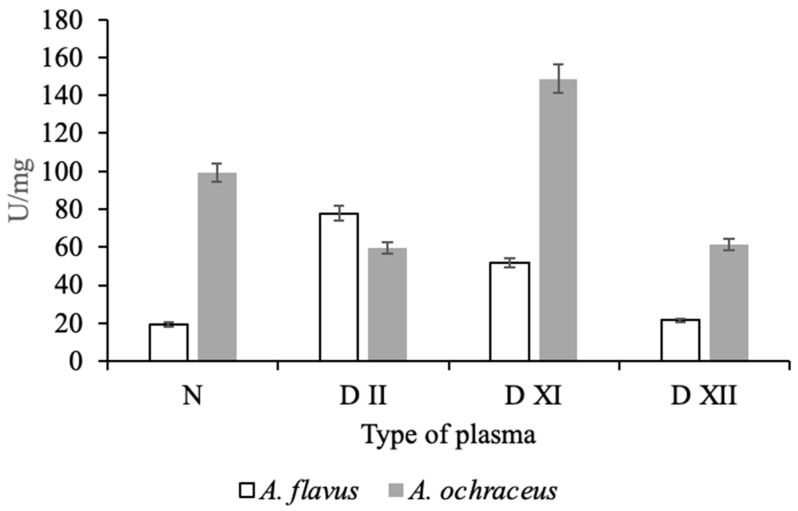
Effect of proteases of *A. flavus* 1 and *A. ochraceus* L-1 on normal and different types of deficient plasmas.

Of considerable interest is the assessment of the severity of the action (specificity) of the isolated micromycete proteases in relation to certain proteins of the plasma hemostasis system, which are responsible for maintaining blood viscosity and the balance between blood coagulation and fibrinolysis. For this, various human blood plasmas, deficient in one of the coagulation factors, were preincubated with proteases from micromycetes. As an analytical signal, the activity of factor X formed as a result of coagulation, one of the key proteins of the hemostasis system, was determined with chromogenic peptide substrate Z-D-Arg-Gly-Arg-pNA. As can be seen from the data presented in Figure 4, when incubated with normal plasma containing all components within the physiological parameters, including factor X, protease *A. ochraceus* L-1 exhibited high activator factor X activity (99.2 U/mg of protein), while protease *A. flavus* 1 exhibited insignificant factor X activity (19.3 U/mg of protein). 

Moreover, the effect of both investigated proteases on deficient plasma is not the same. Protease of *A. flavus* 1 had an effect on plasma deficient in the factors of the external coagulation pathway, factors XI and XII, as well as factor II (prothrombin), as a result of which the formation of factor X may increase. At the same time, protease of *A. ochraceus* L-1 may have contributed directly or indirectly to the activation of factor X during incubation with plasma deficient for factor XI. In experiments with plasma deficient for factors II and XII, the protease activity was lower than in the control (normal plasma). It is known that thrombin (factor IIa) enhances its own accumulation due to the activation of factor XI and factor X through the activation of factors VIII and V, and factor Xa increases its amount in the bloodstream due to the activation of factor XII [33]. In view of the modular-functional structure of the hemostasis system, it is difficult to unambiguously judge the proteins that are targets of *Aspergillus* proteases in blood coagulation reactions; however, it follows from the data obtained that they can be different in both studied strains. This difference can be explained by the specificity of the action of fungal proteolytic enzymes.

It is well known that proteolytic enzymes of *Aspergillus* have a high potential for use as therapeutic agents as components of thrombolytic drugs and in the diagnosis of diseases of the hemostasis system as protein activators of proenzymes [33,34,35]. The obtained data allow us to judge the possibility of proteases of studied strains used as direct thrombolytics capable of cleaving blood clots in a few hours. However, due to the high values of albuminolysis and plasma-coagulating activity, they cannot be the only components of such drugs. Perhaps they should be combined with available anticoagulants and inhibitors, providing a complex effect on blood clots and their environment. However, this requires additional study.

Thus, in this study, we investigated the properties of proteolytic enzymes of two species of *Aspergillus*, *A. flavus* 1 (with a high degree of pathogenicity) and *A. ochraceus* L-1 (a conditional pathogen), to affect various components of the hemostasis system in the case of their penetration into the bloodstream, which serve as one of the means of their pathogenicity and virulence. It was shown that micromycete proteases are highly active in cleaving both globular proteins (albuminolysis) and fibrillar (fibrin), and, to varying degrees, are able to coagulate the plasma of humans and animals (due to proteolysis of factors of the blood coagulation cascade) and are not able to coagulate fibrinogen. The proteases of both *Aspergillus* fully hydrolyze thrombi in 120–180 min. Micromycetes did not show hemolytic activity, but were able to break down hemoglobin.

## Figures and Tables

**Table 1:** Albuminolytic and hemoglobinolytic activity of extracellular proteases of *A. flavus* and *A. ochraceus*.

Protease	Albuminolysis, U_Tyr_/mg of Protein		Hemoglobinolysis, U_Tyr_/mg of Protein
	Human Albumin	Bovine Albumin	
*A. flavus*	482.4	350.8	1064.2
*A. ochraceus*	357.3	603.7	887.2
